# Laser induced white emission and photocurrent of GaN nanoceramics

**DOI:** 10.1038/s41598-025-14109-6

**Published:** 2025-08-07

**Authors:** A. Musiałek, R. Tomala, M. Stefanski, X. Liu, J. Qiu, W. Strek

**Affiliations:** 1 Institute of Low Temperature and Structure Research, Polish Academy of Sciences, Wroclaw, 50422 Poland; 2 State Key Laboratory of Modern Optical Instrumentation, College of Optical Science and Engineering, Zhejiang University, Hangzhou, 310027 China; 3 School of Materials Science and Engineering, Zhejiang University, Hangzhou, 310058 China

**Keywords:** GaN, Nanoceramics, White emission, Photocurrent, Hysteresis loops, Chemistry, Materials science, Optics and photonics, Nanoscale materials

## Abstract

The measurements of the optical properties of GaN nanoceramics are reported. In particular, the laser induced white emission (LIWE) and photocurrent (LIPC) were investigated. LIWE and LIPC was measured in closed cycles: forward, with increasing excitation power density, and backward, with decreasing excitation power density, with hysteresis occurring. The photoconductivity of the obtained nanoceramics was measured at different voltage biases, demonstrating a decrease in initial resistance with applied voltage until saturation was reached, after which no further change occurred at higher voltages. The phenomena are related to charge carrier (electrons) generation via cascade multiphoton ionization and exhibit excitation threshold dependence and non-linear behavior. Simultaneous measurements strongly highlight the connection between both processes. The occurrence of hysteresis suggests potential applications in artificial lighting and/or optical memory devices.

**Supplementary Information:**

The online version contains supplementary material available at 10.1038/s41598-025-14109-6.

## Introduction

Gallium nitride with a band gap 3.4 eV crystallizes in two polytypes: wurtzite, with hexagonal symmetry, and zinc-blende, with cubic symmetry^1^. GaN is a well-known semiconductor characterized by optical and electrical properties such as a wide band-gap, high melting point, thermal conductivity, thermodynamic stability, and high electron mobility^2–8^. Due to these properties, GaN is considered one of the most crucial semiconductors used in light-emitting diodes, photodetectors, solar cells, and devices requiring high frequencies^9^. Luminescence testing of semiconductors is primarily used to analyze existing defects and to obtain information about the crystal quality^10^. As GaN-based devices have led to breakthrough in semiconductor lighting over the last few decades, the properties of both Stokes^7,11,12^ and anti-Stokes^13^ emission are being investigated. The latter results from the phenomenon of laser induced white emission (LIWE) which was first discovered by Wang and Tanner^14^ in single compounds. LIWE results in light close to sunlight emission in the entire visible and near-infrared range. Since then, LIWE has been massively studied in many different inorganic materials^15–22^. It originates from an irradiated spot on the material’s surface generated by a focused CW NIR laser beam, leading to multiphoton ionization. The effect is assisted by electron ejection and photocurrent. An excitation threshold and an exponential increase in emission intensity with a non-linear behaviour characterize both processes. White broadband emission generated by NIR laser is increasingly being analysed as a process related to a photothermal nature^23^. The present research focuses on laser-induced white emission and photoconductivity occurring in GaN nanocrystalline ceramics compacted by using the high-pressure, low-temperature method. According to our best knowledge, up to this moment, there are no reports regarding the hysteresis effect associated with broadband light emission and photocurrent generated by a near-infrared laser simultaneously. Only for LIWE has the dependence of forward and backward power cycles been reported^24^. Moreover, studies on hysteresis have been conducted in the context of magnetism^25^temperature dependent luminescence^26^ or light-induced UV^9^ studies.

## Experimental

The GaN nanocrystalline powder was synthesized using the modified Pechini and ammonothermal methods. The Ga_2_O_3_ powder (99.99%, Thermo Scientific) was added to a beaker and dissolved in concentrated HNO_3_ (65%, POCH Basic) under reflux. Then a citric acid (C_6_H_8_O_7_·H_2_O, 99.4%, POCH Basic) was added to the solution and mixed for 1 h, followed by the addition of ethylene glycol (C_2_H_6_O_2_, 96%, Chempur) and mixing for 1.5 h. The sol was put into the dryer until the resin was formed. After this, the resin was put into the furnace in an air atmosphere at 900 °C for 6 h. The resulting white powder was obtained and placed into a furnace in an ammonia flow at 950 °C for 5 h. The yellow nanopowder was used for sintering ceramics under low temperature high pressure technique. Ceramics were sintered at 500 °C and 8 GPa. Structural properties were confirmed using XRD method using PANalytical X’Pert Pro X-ray powder diffractometer with a Cu radiation source. Optical properties were measured using Agilent CARY 5000 UV-Vis-NIR spectrophotometer, fitted with a Praying Mantis adapter (Harrick) for absorption, FLS980 Edinburgh Instruments apparatus with 360 nm CW laser diode applied as external excitation source for Stokes emission, AVS-USB2000 (Avantes) spectrometer for LIWE in the visible range, and Ocean Optics NIRQuest for LIWE in the near-infrared range. For LIWE in the visible and near infrared region, correction was made, both for the detector sensitivity and for the filters used. For VIS, the FGS0900 filter (Thorlabs Inc.) and for NIR, the FEL1000 filter (Thorlabs Inc.) was used. LIWE experiments have been performed under vacuum conditions (1 × 10^− 5^ mbar) with 975 nm CW laser diode (CNI lasers) applied as an excitation source. The photoconductivity was measured using the Keithley 2400 as a detector and 975 nm CW laser diode (CNI lasers) as an excitation source.

## Results and disscusion

**Fig. 1:**
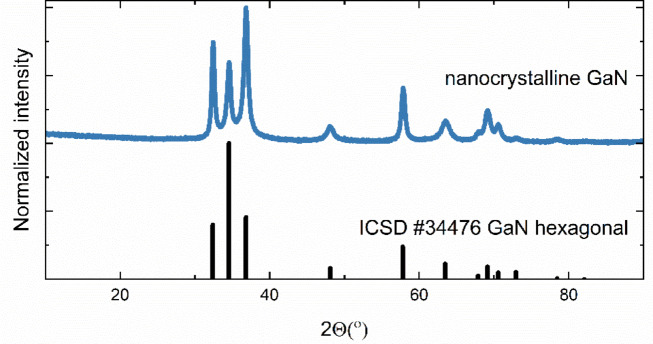
X-ray diffraction pattern of GaN nanocrystals.

The obtained GaN nanocrystals have a hexagonal wurtzite structure, which was confirmed by XRD measurement, shown in (Fig. 1).

**Fig. 2:**
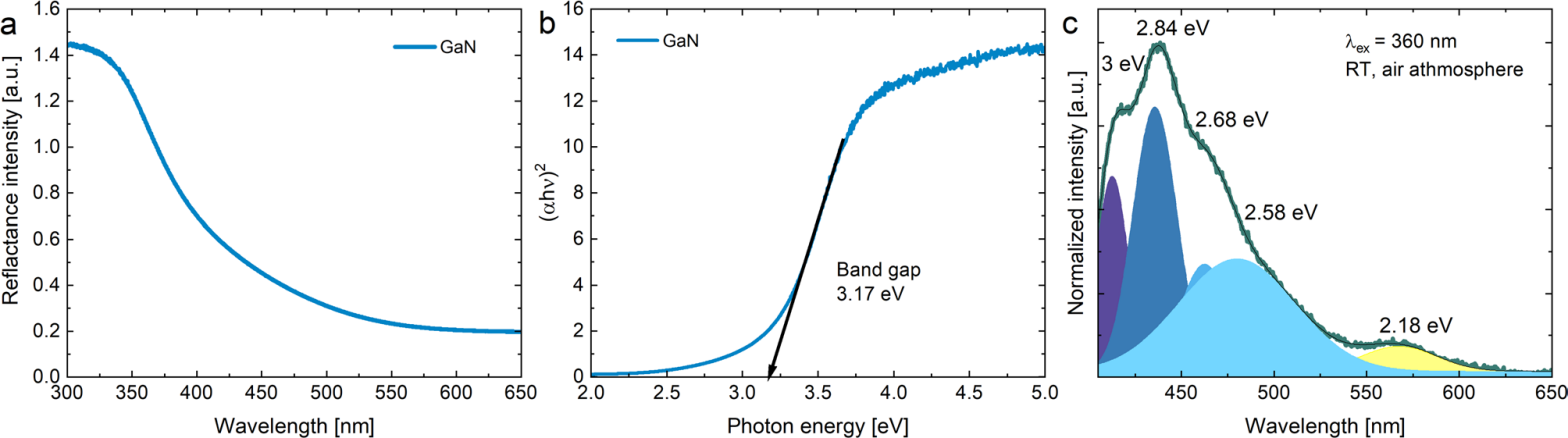
The reflectance absorption spectrum of GaN nanopowder (**a**), the experimental band gap (**b**) and the emission spectrum in the visible range upon λ_exc_ = 360 nm (**c**).

According to the pattern (ICSD #34476), the obtained GaN nanocrystals have a pure structure with the broad reflections indicating nanosize. The average grain size was calculated by Rietveld analysis to be 12 nm. The absorption spectrum of GaN nanocrystalline powder is shown in (Fig. 2). By using the Kubelka-Munk function^27^ the energy band gap was determined to be 3.17 eV. Due to the nano-size of the particles, there are various defects in the structure, such as Ga and N vacancies, surface defects, or grain boundary defects. Therefore, the absorption edge shifts towards lower energies^28^ so the experimental value of the energy gap is smaller than the theoretical one of 3.4 eV^29^.

The wide band emission of GaN nanopowder in a range 405–650 nm with the maximum at 439 nm is shown in (Fig. 2c). Their deconvolution was performed to better understand the nature of the emission bands, resulting in five components with local maxima at 2.18 eV, 2.58 eV, 2.68 eV, 2.84 eV and 3 eV. The more intense bands 2.84 eV and 2.68 eV are most likely associated with V_Ga_ vacancies, and the remaining bands with V_N_ vacancies^28^. The peaks at 3 eV, 2.84 eV are closest to the energy gap value, and may also be related to excitonic recombination^30^. The broad peak at 2.58 eV and the residual peak at 2.68 eV possibly relate to the presence of luminescent centers causing edge shifts for gallium and nitrogen vacancies^31^. The obtained material is also characterized by an undesirable yellow component of luminescence, which is caused by structural defects, indicating the imperfect structure of the obtained material^2,32^.

The research focuses on broadband white light emission (LIWE) characteristics and the accompanying photocurrent phenomenon. First, the studies show that a curve like a hysteresis loop can be observed by measuring the laser power density in the direction forward and backward. So far, two articles have been published attempting to explain the occurrence of hysteresis. These articles focus on carbon materials, diamond^24^and graphene^33^.

**Fig. 3:**
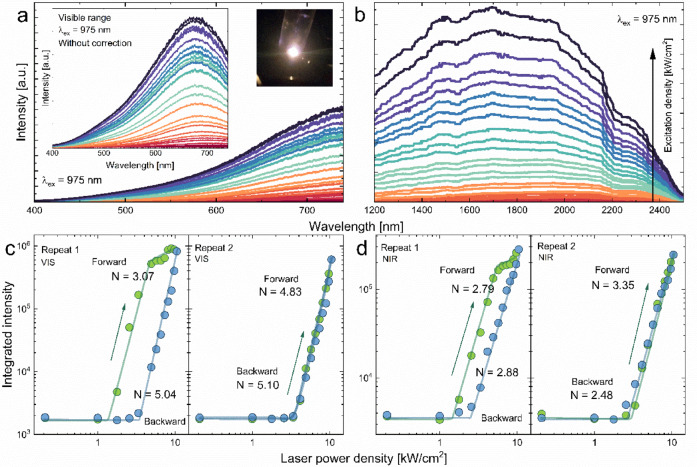
White emission intensity (**a**) near-infrared emission intensity (**b**) for GaN nanoceramics as a function of laser power density. The forward and backward cycles of LIWE in visible region (**c**) and near-infrared region (**d**). Inset the spectrum of LIWE without correction and the image of LIWE of light.

Due to its wide band gap, high thermal conductivity, and chemical stability, GaN is a suitable material for LIWE and photoconductivity investigation. The laser induced white emission spectra measured for GaN nanoceramics for both, visible and near-infrared regions are shown in Fig. 3a, b, respectively. It was characterized by broad bands whose intensity increases exponentially with excitation laser power density and leads to a blueshift of the bands. An increase in LIWE emission intensity starts after crossing the characteristic excitation threshold. The band extends from 400 to 2500 nm with a maximum of 1695 nm. For both regions, the emission threshold is estimated to be slightly above 1 kW/cm^2^. The emission intensity measurements were obtained using two detectors, which is why the emission intensity can be different, and the gap between the visible and near infrared regions results from the use of optical filters and the cutting of the excitation laser beam. Nevertheless, based on these spectra and the N parameter, which in both cases takes similar values, it can be suggested that this is a single broad band. It has also been reported that the two bands measured separately are actually one band^34^. Furthermore, experiments were performed for both regions, initiating hysteresis shape curve by measuring forward with increasing laser power density and then backward with decreasing laser power density. The results are shown in (Fig. 3c,d). In both cases, the characteristic properties of this phenomenon are preserved. The resulting loops are characterized by a threshold value followed by increased emission intensity. Moreover, saturation was demonstrated at high laser power densities. Furthermore, in the case of backward measurements, the N parameter for both ranges has higher values. Additionally, the emission threshold shifts towards higher power densities and saturation does not occur due to the lack of hysteresis. The SI (Figure S1) shows the CIE chromaticity diagram, demonstrating the objective color quality for both anti-Stokes and Stokes emissions. LIWE is characterized by a warm yellow color. Depending on the laser power density, the x and y coefficients locations differ, and as the laser power density increases, it is noticed that the emission color temperature changes towards white.

**Fig. 4:**
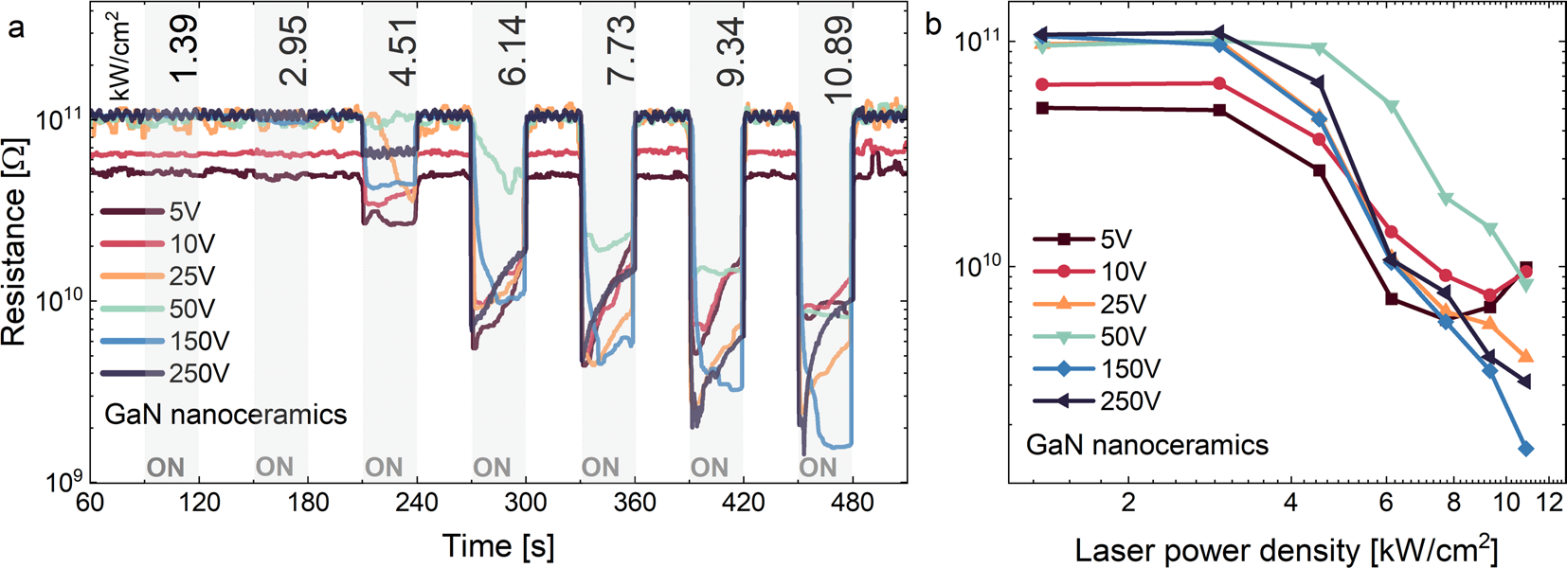
Photoresistance response measured in 30s cycles on/off at different voltage biases (**a**) and resistance dependence (**b**) for GaN nanoceramics.

It was found that the investigated process is assisted by photoconductivity. Therefore, changes in sample resistance were measured depending on the laser power in cycles where the laser was turned on and off every 30 s. The changes were recorded at various voltage biases, as shown in (Fig. 4a). No changes in material resistance were observed at low laser powers, while at higher powers, a decrease in resistance was already observed. This is related to the threshold nature of both white emission and photocurrent. The dependence of resistance on the laser power density was plotted, and the excitation threshold was observed (Fig. 4b). Moreover, the applied voltage affects the initial resistance value. For 5 V, the resistance was the lowest, whereas the highest resistance was observed at 150 V. It should be noted that the resistance was dependent on the applied voltage up to 50 V, and then the saturation was observed. The initial value of resistance is similar for 50 V, 150 V, and 250 V. It can be observed that the photocurrent increases by two orders of magnitude with increasing excitation laser power. The excitation threshold was determined to be about 3 kW/cm^2^ (see Table S1 in SI).

**Fig. 5:**
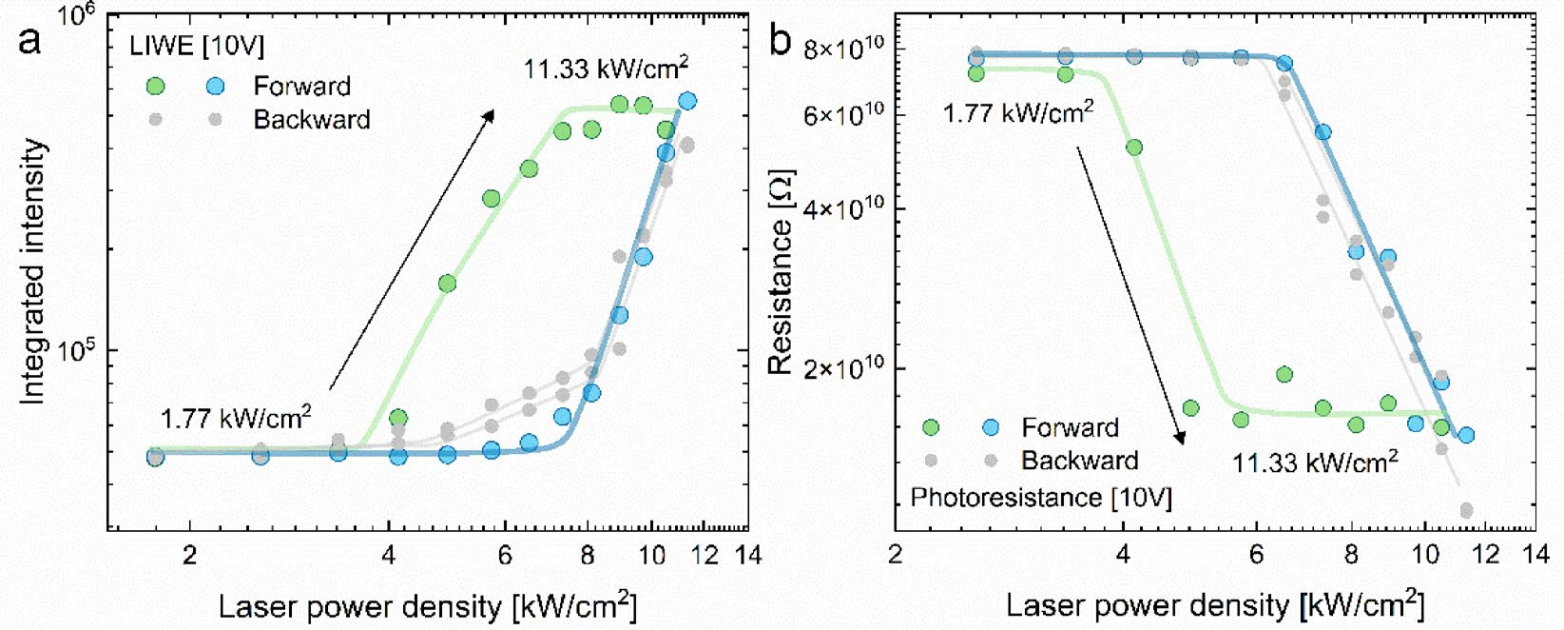
The laser power dependence of integrated intensity of LIWE (**a**) and the maximum of photoresistance of LIPC (**b**) measured in two consecutive measurement cycles: forward and backward (**b**) for GaN nanoceramics.

White emission and photoconductivity were simultaneously performed in laser power density cycles (Fig. 5). The experiments were performed twice in closed (forward and backward) cycles. The differences can be noticed depending on whether the initial laser power density was low or high. For the first one, the hysteresis loop was observed for both LIWE and LIPC processes. However, hysteresis did not occur during measurements from a high initial laser power density.

**Fig. 6:**
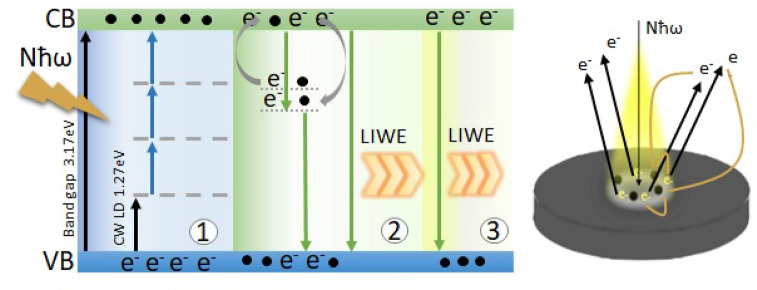
Scheme of proposed mechanism responsible for LIWE divided into 3 stages: (1) Process before threshold value, (2) Process after threshold value including multiphoton ionization and radiative recombination, (3) Saturation process.

The LIWE mechanism has been explained many times, including as blackbody radiation^35,36^ thermal avalanche^14,19,37^ or intervalence charge transfer^38–41^. The characteristics of LIWE are mostly the same, so the mechanism should also be very similar. In this work, to explain the processes taking place, a proposed scheme (Fig. 6.) was used, in which the phenomenon is divided into stages: process before reaching the threshold value, multiphoton ionization and radiative recombination during irradiation after reaching the threshold value and possible occurrence of saturation with a high density NIR laser power.

At low laser power densities, no light is observed until the threshold is exceeded. This is due to the number of photons delivered to the sample surface. GaN nanoceramics is excited with an NIR laser with an energy of 1.27 eV, while the experimental value of the energy gap is 3.17 eV. With the laser power density increase, the probability of photons hitting the same electron increases, ejecting it of the valence band. Then, the multiphoton avalanche process leads to multiphoton ionization on the surface of the tested material. The phenomenon is nonlinear and with the increase of the laser power density, the intensity of emission increases. The dependence of LIWE intensity *I(P)* on the excitation laser power is usually characterized by the power law formula:

1 $$I\left(P\right)\propto\:{P}^{\:\:N}$$

where *I(P)* is the emission intensity, *P* is the excitation laser power, and *N* is related to the number of photons. This equation is used to describe multiphoton absorption transitions. Based on research in our group, where the dependence of power was plotted in different excited spot size it was found that the parameter N cannot be unambiguously treated as the number of absorbed photons. Based on this analysis, one should be more careful in connecting the nonlinearity of the process defined by the parameter N with the actual number of photons participating in the reaction^31,42^. The process of multiphoton ionization (MPI) in irradiated spot at the surface of GaN nanoceramics leads to the broadband white emission (LIWE) assisted by an ejection of hot electrons e^−^ and may be described as:

2 $$GaN + MA\left( {N\hbar \omega } \right) \to GaN^{ + } + ~e^{ - } ~ + {\text{ }}LIWE + NR$$

where MA(*Nħω*) expresses the multiphoton absorption responsible for the multiphoton ionization of the GaN, GaN + is ionized GaN (cathode), LIWE represents laser induced white emission, and NR characterizes the nonradiative quenching and phonon emission processes contributing to the enhancement of thermally active processes. At higher laser power densities, more photons are transmitted to the sample surface, causing electrons to be emitted from the valence band to the conduction band. In addition, a radiative recombination process occurs. An electron from the conduction band recombines with a hole from the valence band, emitting a photon. This process is repeated until all possible emission centres are used. Saturation often occurs. The LIWE still occurs, but its intensity often remains unchanged or decreases. This is probably related to the confinement of some electrons, thus maintaining LIWE. The lack of new emission centres causes a lack of increase in emission intensity.

As explained above, during near-infrared laser irradiation of a sample in a dynamic vacuum, many processes occur, often overlapping. The hysteresis loop is probably formed not only due to photophysical processes but also because of morphological changes. Due to the fact that the measurements were performed depending on the density of the excitation laser beam on nanometric material, changes in the morphology of the sample may have occurred. When the measurement was performed from the lowest laser power, there could have been gradual ionization in GaN, an increase in temperature in the irradiated spot, and changes in morphology after obtaining a high power density. The following changes resulted in a different course when the laser power density decreased. This suggests an irreversible response of the material to the excitation condition. In turn, in the opposite case, the material was first irradiated with high laser power density, probably causing simultaneous ionization and another photophysical process explained in the proposed mechanism, changes in morphology, and high temperature at the beginning. Changing the density to a lower and then to a higher density does not cause changes in the emission intensity, which can confirm the conclusions drawn. Stręk et al.^24^ reported the hysteresis loop behavior for LIWE on diamond material. They explain this as an irreversible process caused by multiphoton ionization. The degree of ionization is related to the irradiation of the sample and the number of ionized atoms. This phenomenon can be used as an effective optical memory. In our case, the confirmation of irreversible process can be repeated measurement of the loop under the same conditions and at the same point, and likewise, the backward cycle, because under high density, the hysteresis loop does not occur. Additionally, based on an article from Zheng et al.23, where an experiment was performed in which two curves were recorded, one for the excitation laser turned on and the other for the excitation laser turned off, a change in the emission intensity was observed, which suggested that the process is not only thermal.

The broadband white light emission phenomenon began to be associated with the photocurrent that appears during sample irradiation. To characterize LIPC, the same formula as in the case of LIWE can be used because both phenomena are nonlinear and exhibit a threshold character. The dependence of photoresistance *R* on laser power density can be expressed by:

3 $$R~\left( P \right)~ \propto P~^{{Npc}}$$

where *N*_*pc*_ is related to the order of multiphoton ionization. The observed drop in resistance after reaching the threshold value is related to photoionization. After the material absorbs infrared photons, charge carriers are created. The higher laser density, the higher number of photons leading to stronger electron-hole recombination and, consequently, higher conductivity.

When discussing the LIWE and photocurrent phenomena, other factors are also considered, with one of the most important being the influence of temperature on the phenomena under study. LIWE was tested in many different luminescent materials, and it can be concluded that the shape of the emission does not depend on the host lattice. However, this phenomenon can be linked to the thermally assisted ionization process and strong optical nonlinearity. The differences resulting from the investigation are mainly associated with a change in the N parameter, depending on the material being tested. Interpretations of this behaviour should be considered. Since the experiments were performed on ceramics, the grains are as close to each other as possible, which reduces the energy localization in the laser spot and increases energy losses due to, for example, heat conduction and changes in the non-linearity of the process^23^. The temperature of emission at different power densities was calculated using the Planck equation (see Table S2 in Supporting Information)

4 $$\:{B}_{\lambda\:}\left(\lambda\:,T\right)=\:\frac{{2hc}^{2}}{{\lambda\:}^{5}}\frac{1}{{e}^{hc/\left(\lambda\:{k}_{B}T\right)}-1}$$

where *h* is Planck’s constant, c is the velocity of light, λ is the wavelength (nm), and *k*_*B*_ is Boltzmann’s constant. The spectrum is dependent on the temperature of the sample. Based on this calculation the temperature was fitting with a good comparison both in relation to the theoretic temperature values associated with blackbody radiation and in relation to the fit of these values in the CIE chromaticity diagram. Despite the good fit of the temperature to Planck’s law, LIWE is mainly caused by the sample ionization process. As shown in articles on other materials, significant differences are observed between the temperature values ​​during LIWE. Using measurements with a thermal camera, the maximum temperature is read at about 1230 K^23^. In the case of other articles, measurements of luminescence nanothermometry, where the emission intensity ratio ^2^H_11/2_ ◊ ^4^I_15/2_ to ^4^S_3/2_ ◊ ^4^I_15/2_ of Er^3+^ ion is used as a temperature probe, the observed maximum temperature takes values ​​of about 900 K^39^. Then, it can be assumed that such differences can be observed in these studies as well, at least because the temperature values based on black body radiation are directly related to a very small point on the sample surface during emission, while the thermal camera shows values where the spot is wider. In connection with this, the temperature distribution is not clearly defined, and the small size of the spot in the laser focus does not allow for direct, accurate measurement without luminescence thermometry. Here one should be careful because, as described above, this method gives values ​​much lower than the blackbody fitting. The question still remains how to properly determine the temperature, such studies are required in the future.

## Conclusions

In this work, the optical and electrical properties of gallium nitride were characterized. GaN with a wurtzite type, hexagonal structure and an average grain size of 12 nm was synthesized. Then, optical characterization including absorption, emission and LIWE was performed. Moreover, electrical changes in GaN during irradiation of the material with a NIR focused laser beam were recorded. From the absorption spectrum, the experimental value of the energy gap (3.17 eV) was calculated. Due to the nano-sized GaN grains, the energy gap is smaller than the theoretical one (3.4 eV). The emission spectrum was recorded and its deconvolution was performed, thus showing that the broad band consists of several emission bands related to both V_Ga_ and V_N_ vacancies and structural defects. The article mainly focuses on the broadband emission of white light induced by a near-infrared laser. The influence of laser power density changes on the emission intensity was investigated. It was found that there is an effective increase in light intensity from the laser power density and a nonlinear nature of the phenomenon being studied. Additionally, it was shown that when measurements were conducted from the lowest laser power density and back, a curve like hysteresis is observed. Nanomaterial was also characterized in terms of photoconductivity. Based on measurements at different applied voltages, changes in the initial resistance value, the nonlinear nature of current changes, and characteristic threshold behavior were observed. The hysteresis shape curves were revealed based on forward and backward measurements of LIWE and LIPC. This confirmed that primarily processes such as LIWE and photocurrent are connected. Probably the processes taking place during material irradiation are multiphoton absorption, ionization of the material and also radiative recombination. A scheme was proposed showing the successive processes occurring in these phenomena. The influence of temperature on LIWE and LIPC was also discussed. Due to the warm light emission, LIWE created on this host can be used for artificial lighting. On the other hand, the occurrence of the hysteresis effect may lead to the use of the obtained material in optical memory effects.

## Supplementary Information

Below is the link to the electronic supplementary material.

Supplementary Material 1

## Data Availability

The data provided in this article are available in Zenodo.org webpage. 10.5281/zenodo.13340674.
