# Supplementary material for: Laser induced white emission and photocurrent of GaN nanoceramics
Source: Sci Rep. 2025 Aug 7;15:28978. doi: 10.1038/s41598-025-14109-6 (PMC12331969; doi:10.1038/s41598-025-14109-6)
Supplement: Supplementary file 1 — Supplementary Material 1 [file 41598_2025_14109_MOESM1_ESM.docx]

Laser induced white emission and photocurrent of GaN nanoceramics

A. Musiałek^1^*, R. Tomala^1^, M. Stefanski^1^, X. Liu^2^, J.Qiu^3^ and W. Strek^1^

^1^Institute of Low Temperature and Structure Research, Polish Academy of Sciences, 50422 Wroclaw, Poland

^2^State Key Laboratory of Modern Optical Instrumentation, College of Optical Science and Engineering, Zhejiang University, Hangzhou 310027, China

^3^School of Materials Science and Engineering, Zhejiang University, Hangzhou 310058, China

**Supporting information**





**Figure S1.** CIE chromaticity diagram of Stokes and Anti-Stokes emission of GaN nanoceramics.

**Table S1.** Dependence of the excitation threshold and LIWE slope of GaN nanocrystalline ceramic on applied voltage.

| GaN | | |
| --- | --- | --- |
| Voltage [V] | Excitation threshold [kW/cm2] | Npc |
| 5 | 0.79 | 2.39 |
| 10 | 0.81 | 2.60 |
| 25 | 0.89 | 2.61 |
| 50 | 1.13 | 2.76 |
| 150 | 1.00 | 3.04 |
| 250 | 1.02 | 2.96 |

**Table. S2.** Temperature dependence of power density. Temperature calculated based of Planck’s formula

| Laser Power [W] | Power density [kW/cm2] | T (K) |
| --- | --- | --- |
| 0.7 | 1.00 | 1320 |
| 0.8 | 1.39 | 1477 |
| 0.9 | 1.77 | 1544 |
| 1 | 2.17 | 1626 |
| 1.1 | 2.56 | 1708 |
| 1.2 | 2.95 | 1746 |
| 1.3 | 3.35 | 1781 |
| 1.4 | 3.74 | 1828 |
| 1.5 | 4.14 | 1886 |
| 1.6 | 4.51 | 1912 |
| 1.7 | 4.91 | 1947 |
| 1.8 | 5.31 | 2008 |
| 1.9 | 5.72 | 2046 |
| 2 | 6.14 | 2076 |
| 2.1 | 6.53 | 2122 |
| 2.2 | 6.91 | 2150 |
| 2.3 | 7.34 | 2170 |
| 2.4 | 7.73 | 2159 |
| 2.5 | 8.11 | 2124 |
| 2.6 | 8.55 | 2145 |
| 2.7 | 8.94 | 2171 |
| 2.8 | 9.34 | 2184 |
| 2.9 | 9.72 | 2200 |
| 3 | 10.12 | 2200 |
| 3.1 | 10.51 | 2224 |
| 3.2 | 10.89 | 2236 |
| 3.3 | 11.33 | 2241 |
